# Quantifying childhood fat mass: comparison of a novel height-and-weight-based prediction approach with DXA and bioelectrical impedance

**DOI:** 10.1038/s41366-020-00661-w

**Published:** 2020-08-26

**Authors:** Mohammed T. Hudda, Christopher G. Owen, Alicja R. Rudnicka, Derek G. Cook, Peter H. Whincup, Claire M. Nightingale

**Affiliations:** Population Health Research Institute, St George’s, University of London, London, UK

**Keywords:** Epidemiology, Epidemiology

## Abstract

Accurate assessment of childhood adiposity is important both for individuals and populations. We compared fat mass (FM) predictions from a novel prediction model based on height, weight and demographic factors (height–weight equation) with FM from bioelectrical impedance (BIA) and dual-energy X-ray absorptiometry (DXA), using the deuterium dilution method as a reference standard. FM data from all four methods were available for 174 ALSPAC Study participants, seen 2002–2003, aged 11–12-years. FM predictions from the three approaches were compared to the reference standard using; *R*^2^, calibration (slope and intercept) and root mean square error (RMSE). *R*^2^ values were high from ‘height–weight equation’ (90%) but lower than from DXA (95%) and BIA (91%). Whilst calibration intercepts from all three approaches were close to the ideal of 0, the calibration slope from the ‘height–weight equation’ (slope = 1.02) was closer to the ideal of 1 than DXA (slope = 0.88) and BIA (slope = 0.87) assessments. The ‘height–weight equation’ provided more accurate individual predictions with a smaller RMSE value (2.6 kg) than BIA (3.1 kg) or DXA (3.4 kg). Predictions from the ‘height–weight equation’ were at least as accurate as DXA and BIA and were based on simpler measurements and open-source equation, emphasising its potential for both individual and population-level FM assessments.

## Introduction

With the ongoing childhood obesity epidemic in many countries including the United Kingdom (UK), accurate assessment of childhood adiposity is important both for individual and population level assessment. Body mass index (BMI), an indirect and widely used marker of adiposity, has serious limitations in childhood populations [1–3]. Crucially, as a weight-based measure, it does not discriminate between fat mass (FM) and fat-free mass, which can vary markedly in individuals with a given BMI [2]. More direct methods of FM assessment may represent an important advance in the assessment of adiposity, including dual energy x-ray absorptiometry (DXA) and bioelectric impedance analysis (BIA) [3], which are increasingly available and are being used more frequently but may lack precision [3–5]. We recently developed and validated an alternative approach for FM assessment, the ‘height–weight equation’ (Supplementary Box 1), which accurately estimated FM from simple measurements of height and weight combined with information on sex, age and ethnicity [6]. The ‘height–weight equation’, derived in a large dataset of UK children aged 4–15 years, demonstrated high levels of predictive accuracy upon internal and external validation [6]. Here we compare the accuracy of FM predictions from the ‘height–weight equation’ with FM obtained from BIA and DXA, using the deuterium dilution (DD) method as a reference standard, in a study of UK children aged 11–12 years.

## Research design and methods

The Avon Longitudinal Study of Parents and Children (ALSPAC) is a UK-based birth cohort study containing detailed assessments from 14,062 live born children, and their mothers, from the Bristol area between April 1991 and December 1992, including information on height, weight, sex, ethnicity, and age [7, 8]. From the full cohort, described in Appendix 1, a subsample of 176 children were recruited, stratified by sex and BMI to be representative of the cohort, and underwent detailed body composition assessments using the DD, BIA and DXA approaches and measures of height and weight taken at 11–12 years between 2002 and 2003 [5]. Height measurements were made using the Harpenden Stadiometer. Weight and BIA assessments were made using the Tanita TBF305 foot–foot BIA, entering height data manually, and using manufacturer’s software to obtain FM estimates [9]. Whole body DXA scans were conducted using a Lunar Prodigy fan-beam densitometer using paediatric software to obtain FM, as described previously [9], where the child’s height, weight, date of birth, gender and ethnicity (if available) were inputted into the machine before the scan. Ethnicity was based on parental self-reported information. DD assessment involved estimating fat-free mass (and indirectly FM) from total body water. Participants provided saliva samples before consuming water containing deuterium oxide, and also 4–5 h after. This allowed for the analysis of the exchange of deuterium in body water using infrared spectroscopy [10]. Please note that the study website contains details of available data through a searchable data dictionary and variable search tool: http://www.bristol.ac.uk/alspac/researchers/our-data.

Statistical analyses were performed using Stata v15. The ‘height–weight equation’ (Supplementary Box 1), based on measurements of height, weight, sex, age and ethnicity, was used to predict FM within the study population as described in the original publication [6]. All individuals with complete and non-negative measurements of FM from all four approaches comprised the analysis sample; median values of key variables are presented, by sex and overall, in Supplementary Table 1. The predictive performance of the three approaches (the ‘height–weight equation’, DXA and BIA) were assessed and compared, using the fourth approach, the DD method, as the reference standard, based on the following established performance metrics [11] and their respective ideal values: (i) *R*^2^—percentage of the variance in reference standard FM explained by predicted FM; (ii) calibration (slope and median-centred-intercept)—agreement between predicted and reference standard FM assessed in terms of the slope (ideal value being 1) and median-centred-intercept (ideal value being 0) from a simple linear regression model regressing reference standard FM on predicted FM values (FM values were centred around the median FM value to allow for meaningful interpretation of the intercept of agreement at the median FM level), and (iii) root mean square error (RMSE)—average difference between predicted and reference standard FM values (lower values indicating more accurate predictions). The overall calibration of each approach was assessed graphically by plotting agreement between median predicted and median reference standard FM within tenths of predicted FM. Sensitivity analyses were conducted to: (i) assess the sex-specific predictive performance of each method and (ii) to assess the impact of using the an alternative published BIA equation [12] to obtain FM estimates, rather than using manufacturers equations.

## Results

There were 176 children with complete measurements of FM from all four methods. Two children had negative FM values from DXA assessments, leaving 174 children included in the final analysis. The characteristics of the study population are presented in Supplementary Table 1. The average age was 11.8 years (range: 11.5–12.8 years) and median FM from the DD reference method was 9.4 kg (IQR: 7.0).

**Fig. 1:**
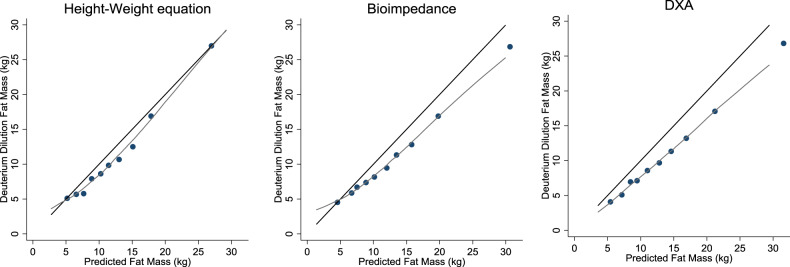
Calibration plot of reference standard fat mass and predicted values, across tenths of predicted fat mass. Black line represents line of equality. Grey line represents a local regression smoother through individual level data points.

The predictive performance metrics from the three approaches, compared with DD, are presented in Table 1. The ‘height–weight equation’ produced an *R*^2^ value of 89.8% (95% CI: 86.9, 92.7%) and a RMSE of 2.6 kg (Table 1). In comparison, the *R*^2^ value from DXA was 94.8% (95% CI: 93.3, 96.3%) with a RMSE of 3.4 kg and from BIA assessments the *R*^2^ value was 91.0% (95% CI: 88.4, 93.5%) with a RMSE of 3.1 kg. These RMSE values correspond to an average proportion of error of 28% of median FM from the ‘height–weight equation’, 33% from BIA and 36% from DXA. Whilst the median-centred-intercepts were similar and close to the ideal of 0 from each of the approaches, (‘height–weight equation’: −0.05 kg [95% CI: −0.40, 0.30 kg]); DXA: −0.24 kg [95% CI: −0.49, 0.01 kg]; BIA −0.07 kg [95% CI: −0.39, 0.26 kg]), the slope from the ‘height–weight’ equation of 1.02 (95% CI: 0.97, 1.08) was closer to the ideal value of 1 than those of DXA (slope = 0.88 [95% CI: 0.85, 0.91] and BIA (slope = 0.87 [95% CI:0.82, 0.91]). Overall calibration, assessed across tenths of predicted FM, demonstrates the overall agreement between reference standard and predicted FM from the three approaches (Fig. 1; Supplementary Table 2) across the range of FM values. Agreement between predicted and reference standard FM at the lower end of the FM distribution was accurate and generally similar for all three methods. FM predictions at the upper end of the FM distribution were accurate from the ‘height–weight equation’, but less so from BIA and DXA (Fig. 1; Supplementary Table 2). For example, in the lowest decile, the differences between median reference standard and predicted FM were −0.08, −0.04 and −1.34 kg from the ‘height–weight equation’, BIA and DXA, respectively. However, in the top decile, respective differences were 0.01, −3.78 and −4.77 kg (Supplementary Table 2).

**Table 1:** Predictive performance statistics from three approaches, compared with reference standard deuterium dilution assessments of fat mass.

FM prediction method	Overall [*N* = 174]			
	*R*^2^ (%)	Calibration Slope	Calibration intercept (kg)	RMSE (kg)
Height–weight equation	89.8 (86.9, 92.7)	1.02 (0.97, 1.08)	−0.05 (−0.40, 0.30)	2.59
BIA	91.0 (88.4, 93.5)	0.87 (0.82, 0.91)	−0.07 (−0.39, 0.26)	3.05
DXA	94.8 (93.3, 96.3)	0.88 (0.85, 0.91)	−0.24 (−0.49, 0.01)	3.40

Calibration slope and intercept based on FM values centred around the median FM. Ideal values of calibration slope and intercept are 1 and 0, respectively.

*RMSE* root mean square error.

Sensitivity analyses assessing the predictive performance of the methods separately for boys and girls showed a similar pattern of results to the sex-combined analyses (Supplementary Table 3 and Supplementary Fig. 1). Although there is a systematic difference in FM between boys and girls, the predictive performance of the model is unaffected by sex. Furthermore, utilising the alternative BIA equation [12] to assess the impact on the accuracy of FM predictions provided similar results to those obtained using the manufacturer’s software, with an *R*^2^ value of 91.9% (95% CI: 89.7, 94.2%), a calibration slope of 0.88 (95% CI: 0.84, 0.92) with a median-centred-intercept of 0 kg (95% CI: −0.31, 0.30) and a RMSE value of 2.3 kg.

## Discussion

This study compared the predictive performance of a novel approach to assessing childhood FM based on measurements of height, weight and simple demographic information with FM predictions obtained from two other methods; BIA and DXA, using the DD method as a reference standard, in a study of children aged 11–12 years. Amongst 11–12-year-old children, the ‘height–weight equation’ had a calibration slope close to the ideal of 1, a median-centred-intercept close to the ideal of 0 and provided FM estimates with the lowest RMSE (average individual level error). DXA and BIA also provided predictions with low individual level error, but suffered from overall mis-calibration demonstrated by the decreased accuracy of FM predictions with increasing FM, suggesting that DXA and BIA may be less suited to children with increasing adiposity. This is due to the effect of an accurate calibration intercept combined with a mis-calibrated slope, demonstrating the need to consider these two metrics together.

The results of this study are consistent with findings of other validation studies carried out to assess the performance of DXA or BIA using either the DD method [5, 13, 14] or other multi-component models [4, 15, 16] as reference methods. These studies similarly reported that although, on average, FM (expressed either as kilograms of fat or FM%), was overestimated by DXA [4, 5, 13, 15, 16] and BIA [5, 14], the accuracy of DXA and BIA assessments varied considerably across the range of FM values, with DXA over-estimating FM at higher levels and under-estimating at lower levels [5, 13, 16]. Finally, the study by Sopher et al. also showed that DXA estimates of FM% explained a high proportion of the variation in FM% from the reference method.

This study provides a comparison of three approaches to estimate childhood FM providing an independent and comparable assessment of their respective predictive performances. However, whilst the sample size of 174 is sufficient to provide reasonable precision of the predictive performance metrics for the current analyses, participants were drawn from a narrow age range and a single ethnic group, making wider generalisability speculative. Whilst the estimates of FM from BIA were obtained using the manufacturer’s equations, results were consistent when using an alternative BIA equation to estimate FM. Furthermore, just as new BIA equations or DXA software are being developed, the ‘height–weight’ equation can also be updated as more data become available. Although data collection was undertaken between 2002 and 2003, the dataset used in this study to compare the predictive performance from each approach, contains children with a wide range of anthropometric characteristics, which remain reasonably consistent with measures from more contemporary children [17].

The findings of this study have important implications for clinical practice and preventive policy in the UK and similar populations. The ‘height–weight equation’, which is an open-source equation and based on readily available measurements, predicts FM levels at least as accurately as DXA and BIA, which rely on both costly equipment and manufacturer’s software/equations which are typically not openly available. Due to the high level of accuracy in predicting FM obtained by the ‘height–weight equation’, it is likely to prove a more effective use of height and weight information than the use of indirect markers of adiposity, such as weight-for-height indices (e.g. BMI). This approach, after validation and possibly re-calibration, could also be beneficial in low-income populations with emerging increased concerns about overweight, and where the costs of more sophisticated assessments of FM may remain prohibitive.

## Supplementary information

SUPPLEMENTAL MATERIAL

Appendix
