# Supplementary material for: Quantifying childhood fat mass: comparison of a novel height-and-weight-based prediction approach with DXA and bioelectrical impedance
Source: Int J Obes (Lond). 2020 Aug 26;45(1):99–103. doi: 10.1038/s41366-020-00661-w (PMC7752759; doi:10.1038/s41366-020-00661-w)
Supplement: Supplementary file 1 — SUPPLEMENTAL MATERIAL [file 41366_2020_661_MOESM1_ESM.docx]

**SUPPLEMENTARY MATERIAL - Quantifying childhood fat mass: comparison of a novel height-and-weight-based prediction approach with DXA and bioelectrical impedance**

SUPPLEMENTARY TABLE 1: CHARACTERISTICS OF THE STUDY PARTICIPANTS

|  | Median (LQ - UQ) | | |
| --- | --- | --- | --- |
|  | Males (N=84) | Females (N=90) | Overall (N=174) |
| Age (years) | 11.9 (11.8 – 12.0) | 11.8 (11.8 – 12.0) | 11.8 (11.8 – 12.0) |
| Height (cm) | 150.7 (146.1 – 156.3) | 152.0 (147.2 – 155.9) | 151.9 (146.6 – 156.2) |
| Weight (kg) | 42.5 (36.0 – 47.5) | 44.7 (38.6 – 50.8) | 43.2 (37.2 – 50.2) |
| White ethnic origins (N, %) | 78 (93) | 82 (91%) | 160 (92%) |
| DD fat mass (kg)* | 7.8 (5.6 – 12.6) | 10.5 (7.4 – 13.8) | 9.4 (6.5 – 13.5) |

Abbreviations: DD = deuterium dilution method

| Tenth of predicted fat mass | Height-Weight Equation | | BIA | | DXA | |
| --- | --- | --- | --- | --- | --- | --- |
|  | Median Reference standard | Median Predicted | Median Reference standard | Median Predicted | Median Reference standard | Median Predicted |
| 1 | 5.1 | 5.2 | 4.5 | 4.6 | 4.1 | 5.4 |
| 2 | 5.7 | 6.6 | 5.9 | 6.7 | 5.1 | 7.2 |
| 3 | 5.8 | 7.7 | 6.7 | 7.5 | 7.0 | 8.5 |
| 4 | 7.9 | 8.9 | 7.4 | 8.9 | 7.1 | 9.5 |
| 5 | 8.6 | 10.3 | 8.2 | 10.1 | 8.6 | 11.0 |
| 6 | 9.8 | 11.4 | 9.5 | 12.0 | 9.7 | 12.8 |
| 7 | 10.7 | 13.0 | 11.3 | 13.5 | 11.3 | 14.6 |
| 8 | 12.5 | 15.1 | 12.8 | 15.8 | 13.2 | 16.9 |
| 9 | 16.9 | 17.9 | 16.9 | 19.8 | 17.1 | 21.2 |
| 10 | 27.0 | 27.0 | 26.9 | 30.6 | 26.8 | 31.6 |

SUPPLEMENTARY TABLE 2: AVERAGE VALUES OF REFERENCE STANDARD FAT MASS AND PREDICTED FAT MASS, ACROSS TENTHS OF PREDICTED FAT MASS FROM EACH OF THE THREE APPROACHES

FOOTNOTE: Tenths of predicted fat mass formed independently for each method. Median values of both reference standard fat mass and predicted fat mass were calculated within each decile group.

SUPPLEMENTARY TABLE 3: SEX-SPECIFIC PREDICTIVE PERFORMANCE STATISTICS FROM EACH OF THE THREE APPROACHES, COMPARED WITH REFERENCE STANDARD DEUTERIUM DILUTION ASSESSMENTS OF FAT MASS

|  |  | **R^2^ (%)** | **Calibration Slope** | **Calibration intercept (kg)** | **RMSE (kg)** |
| --- | --- | --- | --- | --- | --- |
| **Males**  **[N=84]** | height-weight equation | 87.9 (83.1, 92.8) | 1.05 (0.97, 1.14) | 0.30 (-0.18, 0.79) | 2.28 |
|  | BIA | 88.4 (83.8, 93.1) | 0.85 (0.79, 0.92) | 0.19 (-0.28, 0.67) | 2.82 |
|  | DXA | 95.1 (93.1, 97.1) | 0.85 (0.81, 0.89) | -0.25 (-0.56, 0.07) | 3.00 |
|  |  |  |  |  |  |
| **Females**  **[N=90]** | height-weight equation | 91.5 (88.1, 94.8) | 1.04 (0.97, 1.10) | -0.10 (-0.59, 0.38) | 2.86 |
|  | BIA | 92.2 (89.1, 95.3) | 0.88 (0.82, 0.93) | -0.14 (-0.61, 0.32) | 3.25 |
|  | DXA | 94.8 (92.7, 96.9) | 0.91 (0.87, 0.96) | -0.18 (-0.56, 0.20) | 3.73 |

Footnote: RMSE = Root mean square error. Calibration slope and intercept based on FM values centred around the median FM. Ideal values of calibration slope and intercept are 1 and 0, respectively.

Supplementary Box 1: THE ‘HEIGHT-WEIGHT EQUATION’ FOR THE PREDICTION OF FAT MASS IN CHILDREN

$$Fat Mass= weight-\exp[0.3073* {height}^{2}-10.0155*{weight}^{-1}+0.004571*weight+0.01408*BA - 0.06509*SA-0.02624*AO-0.01745*Other-0.9180*\ln\left( age \right)+0.6488*{age}^{0.5}+0.04723*male +2.8055]$$

exp = exponential function, ln = natural logarithmic transformation.

BA, SA, AO and Other = 1 if child is of Black, South Asian, Other Asian, or Other ethnic origins respectively and = 0 if not.

If child is of unknown ethnic group, treat as of White ethnic origins.

Height is measured in metres, weight in kilograms, age in years and fat mass in kilograms.

SUPPLEMENTARY FIGURE 1: CALIBRATION PLOT OF REFERENCE STANDARD FAT MASS AND PREDICTED VALUES, ACROSS TENTHS OF PREDICTED FAT MASS, BY SEX

Footnote: Black line represents line of equality. Grey line represents a local regression smoother through individual level data points
